# Editorial: Case reports in predictive toxicology: 2022

**DOI:** 10.3389/fphar.2023.1180949

**Published:** 2023-04-03

**Authors:** Eleonore Fröhlich, Dirk Steinritz

**Affiliations:** ^1^ Center for Medical Research, Medical University of Graz, Graz, Austria; ^2^ Bundeswehr Institute of Pharmacology and Toxicology, Munich, Germany

**Keywords:** case reports, toxicology, adverse drug effects, toxicants exposure, lung cancer

Toxicity in humans may occur by exposure to natural (toxins) and manufactured subtances and products (toxicants). These substances cause damage to the organism either by contact with epithelial barriers such as mucosae and skin or by the parenteral route (inhalation or ingestion). In addition to these substances with exclusively negative effects on the human body, there are molecules and devices used in therapeutical treatment in which toxicity represents an unwanted side effect. Toxicity may be acute, occurring after one exposure, or (sub)chronic, after repeated exposure for durations ranging from weeks to years. To identify such effects, guidelines have been published by the Organisation for Economic Co-operation and Development (OECD) for chemicals and by national pharmaceutical agencies, such as the Food and Drug Administration (FDA) in the United States, for preclinical drug testing. These guidelines are used for a systematic evaluation of the toxic effects that an agent can produce and comprise several administration routes, exposure times and repetition, and specific aspects of toxicity (sensitization, reproduction, genotoxicity, *etc.*). Testing is performed typically in two mammalian species, but this is expected to change in the future due to the high rate of incorrect assessments and animal welfare concerns. In an evaluation of 108 antitumor agents, the positive predictive value of toxic effects obtained in preclinical studies with mouse, rat, dog, and monkey was 0.65, and the negative predictive value was 0.5 for human toxicity (Atkins et al., 2020). It was therefore not unexpected that the majority of drug compounds should fail in later clinical trials. This failure occurred at a rate of 85% in the early clinical trials, but also only half of those that survived until phase III were approved for clinical use (Mak et al., 2014).

Even when a drug is on the market and an absence of adverse effects is demonstrated in premarketing randomized controlled trials (RCTs) and various postmarketing surveillance (PMS) studies (Suvarna, 2010), toxicity can occur. Functional polymorphism in metabolizing enzymes and differences in organ function and diseases influence the individual reaction to chemical compounds. A Monte Carlo simulation has shown a variation of dieldrin ingestion across age, sex, and race/ethnicity by a factor of six, resulting in an increase in the probability of occurrence of adverse health effects by a factor of seven in a highly susceptible population (Li and Li, 2021). Case studies are a good way to raise the awareness of unexpected toxicities and may provide useful information for a safe treatement.

This Research Topic comprises five articles, including three that report unexpected (in terms of the manifestation or extent) drug toxicities and two that report toxicities due to overdoses. The checkpoint inhibitor pembrolizumab was given to a patient with large-cell lung carcinoma, and multiorgan failure with fulminant myocarditis was observed. The treatment with a combination of high steroid doses and implantation of a cardiac pacemaker was successful. Since immune-related adverse effects are not uncommon in treatment with checkpoint inhibitors, the authors suggested the introduction of predictive markers Xie et al.

In a patient with stage IV anaplastic lymphoma kinase-rearranged non-small-cell lung cancer who was receiving alectinib therapy, drug-induced pneumonia with indolent onset was diagnosed. After discontinuation of alectinib and treatment with steroids, the objective and subjective findings improved. The take-home message from this report is that drug-induced pneumonia by alectinib could have a late onset Chen et al.

The multikinase inhibitor sorafenib is known for its potential to produce rash and desquamation as adverse effects. However, in a reported case of a patient with unresectable hepatocellular carcinoma, the severity was unusually high (grade 3). After discontinuation of sorafenib therapy combined with steroid infusion and piperacillin/tazobactam treatment, the skin lesions disappeared. Despite the particularly severe manifestation, the recommended treatment with steroids and antibiotics was successful Lin and Liu.

In two studies that examine single cases of overdose, one deals with the ingestion of the organophosphate pirimiphos with suicidal intention and the other with an unexpected reaction to the antiarrhythmic drug pilsicainide. Pirimiphos, a known inhibitor of acetylcholine esterase, was ingested in combination with alcohol by the patient, who had Child–Pugh class 2 alcoholic liver cirrhosis with several co-morbidities. After treatment with obidoxime and atropine, the situation improved but remained critical for 4 days. The decreased metabolic capacity of the liver resulted in high pesticide levels without severe cholinergic crisis. The authors illustrated the problem of delayed cholinergic crisis onset, which, however, could be managed by the delayed administration of the antidote Zellner et al.

In the case of a recorded accidental death by the generally safe antiarrhythmic drug pilsicainide, polypharmacy may have been the cause, because verapamil, furosemide, warfarin, acetaminophen, ephedrine, and methylephedrine, in addition to pilsicainide, were detected in the blood. It is assumed that pilsicainide may have pro-arrhythmic effects in case of an overdose Takei et al.

The presented reports show only a small part of the spectrum of potential toxic reactions caused by toxicants. Compared to toxicity screening, the findings of case studies may be relevant only for a small proportion of patients. Despite this, it is important to document these findings, because they might otherwise go unnoticed.
